# Induced chromosomal instability in triple negative breast cancer cells promotes M2-like polarization of macrophages

**DOI:** 10.1038/s41598-026-55242-0

**Published:** 2026-06-06

**Authors:** Ruifang Tian, Siqi Zheng, Floris Foijer

**Affiliations:** 1 European Research Institute for the Biology of Ageing, University Groningen, University Medical Center Groningen, NL 9713 AV Groningen, the Netherlands; 2 Department of Oncology, Hunan Provincial People’s Hospital (The First Affiliated Hospital of Hunan Normal University), Hunan Hepatobiliary and Pancreatic Cancer Clinical Medical Research Center, Key Laboratory of Study and Discovery of Small Targeted Molecules of Hunan Province, 61 West Jiefang Road, Furong District, Changsha, 410006 Hunan China; 3 Institut Jules Bordet, 90 Rue Meylemeersch, Level 4, 1070 Brussels, Belgium

**Keywords:** Chromosomal instability, Tumor-associated macrophages, Tumor microenvironment, Migration, Triple-negative breast cancer, Cancer, Immunology

## Abstract

Chromosomal instability (CIN) is a defining feature of triple-negative breast cancer (TNBC) and is increasingly recognized as a driver of tumor evolution and immune escape. However, how CIN^HIGH^ tumor cells modulate innate immune cells, particularly macrophages, remains unclear. Here, we used the MPS1 inhibitor reversine to induce acute CIN in the TNBC cell line MDA-MB-231 and examined its impact on human macrophage polarization and function. Reversine-induced CIN stably increased the expression of macrophage-attracting cytokines, including CCL2, CCL5, and IL-6. In direct co-cultures, reversine-treated cancer cells promoted M2-like macrophage polarization, accompanied by a modest increase of M1-like markers, whereas in non-contact transwell co-cultures, soluble paracrine signals from reversine-treated TNBC cells only promoted M2-like polarization. Furthermore, macrophages exposed to reversine-treated TNBC cells showed enhanced uptake of cancer cells, further underscoring their activation by cells with reversine-induced CIN. Finally, we found that macrophage activation coincided with the secretion of factors that promoted cancer cell migration, suggesting that immune-cancer cell interaction promotes metastasis. Together, our findings show that drug-induced CIN in TNBC cells promotes polarization of co-cultured macrophages, promoting an immunosuppressive, pro-migratory niche that may be exploitable for therapies targeting CIN-associated immune modulation.

**Supplementary Information:**

The online version contains supplementary material available at 10.1038/s41598-026-55242-0.

## Introduction

Triple-negative breast cancer (TNBC) is an aggressive subtype of breast cancer that lacks expression of the estrogen receptor (ER), progesterone receptor (PR), and HER2. TNBC is highly heterogeneous, has limited treatment options, and is associated with a poor prognosis^1^. Therefore, new treatment targets are urgently needed.

Chromosomal instability (CIN) is an increased rate of chromosome segregation errors during mitosis, resulting in aneuploidy, and affects more than 80% of human cancers^2–4^. Indeed, the vast majority of TNBCs display CIN and tumor heterogeneity, ultimately leading to metastasis, drug resistance, thus yielding a poor prognosis^5–7^. While CIN typically compromises the viability of non-cancer cells and is highly immunogenic^8^, paradoxically, clinical data indicate that tumors with high levels of CIN commonly display immunosuppression^9–13^, suggesting that CIN^HIGH^ cancers have developed mechanisms to evade immune recognition.

The tumor microenvironment (TME) is increasingly recognized as a key player in tumor progression and therapy response. It consists of various stromal cell types, including fibroblasts, endothelial cells, and immune cells. Among the latter cell type, macrophages are one of the most abundant innate immune cells and can exhibit distinct phenotypes including pro-inflammatory, anti-tumor M1-like, but, conversely, also immunosuppressive, tumor-promoting M2-like states^14,15^. In most solid tumors, tumor-associated macrophages (TAMs) are skewed toward the M2-like phenotype, which contributes to immunosuppression and tumor progression^16–18^. A comprehensive bioinformatic analysis of aneuploid tumors revealed that highly aneuploid tumors display a decreased ratio of M1- to M2-associated gene expression^9^, further supporting a potential connection between CIN and macrophage polarization.

Given the plasticity of TAMs, strategies aimed at reprogramming macrophage polarization are receiving increasing interest as potential immunotherapeutic interventions^19–21^. However, whether and how CIN phenotypes in TNBC influence TAM polarization dynamics remains poorly understood.

In this study, we determine whether inducing acute CIN in TNBC cells—on top of their inherent chronic CIN—influences macrophage behavior. Using MDA-MB-231 TNBC cells in combination with the CIN-inducing MPS1 inhibitor reversine, we establish a TNBC cell model to study the impact of CIN on macrophage polarization. We find that reversine-induced CIN upregulates chemokine expression in TNBC cells and that exposed macrophages predominantly exhibit an M2-like phenotype with a minor induction of M1-like markers. Furthermore, co-cultured macrophages display enhanced phagocytic uptake. Similarly, in non-contact co-cultures of TNBC cells and macrophages, we also find that indirectly exposed macrophages primarily display an M2-like polarization. Finally, we find that TNBC cells in co-cultures display enhanced migration, suggesting that interactions within the microenvironment might contribute to metastatic behavior of cancer cells. Together, these data suggest that, while direct interaction between TNBC cells with reversine-induced CIN and M0 macrophages might lead to some M1-like polarization, M2-like polarization is the most common outcome when macrophages are exposed to TNBC cells with induced CIN. We conclude that reversine-induced CIN promotes macrophage polarization and increased migration, revealing another potential route of how cancer cells with CIN influence their microenvironment towards an immunosuppressive promigratory state.

## Results

### Establishing a CIN^HIGH^ cancer cell–macrophage co-culture model

**Fig. 1:**
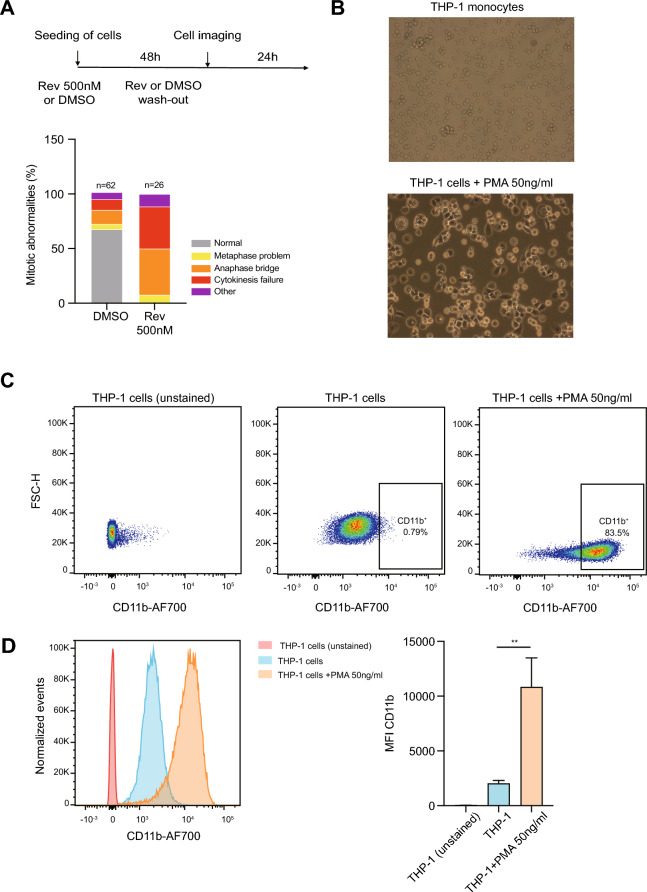
Establishment of a reversine-induced TNBC model and differentiation of THP-1 cells into M0 macrophages. (**A**) Schematic overview of reversine treatment regimen followed by quantification of mitotic abnormalities through live-cell imaging. (**B**) Representative phase-contrast images showing THP-1 cell morphology before and after treatment with 50 ng/ml PMA for macrophage differentiation. (**C**) Flow-cytometric scatter plots of CD11b expression in THP-1 cells before and after PMA-induced differentiation into M0 macrophages. (**D**) Flow histogram and quantification of CD11b mean fluorescence intensity (MFI) from (C). Data represent mean ± SD from three independent biological replicates. Statistical significance: *p < 0.05; **p < 0.01; ***p <  0.001; ****p < 0.0001 (unpaired two-tailed Student’s t-test).

To investigate the impact of chromosomal instability (CIN) on macrophage behavior in triple-negative breast cancer (TNBC) cells, we first established a protocol to induce acute CIN in TNBC cells. For this, we selected the MDA-MB-231 TNBC cell line, which exhibits low basal CIN levels, and treated them with the MPS1 inhibitor reversine to induce acute CIN^8^. Live cell time-lapse imaging was used to confirm and quantify CIN^22^. While approximately 25% of vehicle-treated cells exhibited chromosome segregation errors during mitosis (Fig. 1A), nearly all reversine-treated cells (500 nM) exhibited chromosome segregation abnormalities after 48 h, indicating a marked increase in CIN (Fig. 1A).

To establish a human macrophage model, we used the THP-1 cell line, derived from acute monocytic leukemia. This cell line is widely used to study monocyte-macrophage differentiation, function, and related signaling pathways^23^. We first induced THP-1 cells to differentiate into unpolarized M0 macrophages using Phorbol Myristate (PMA)^24^. THP-1 cells were treated with 50 ng/ml PMA for 48 h, followed by a 24-h rest period. While THP-1 cells remained non-adherent, PMA-treated cells attached accompanied by an increased cell size (Fig. 1B)^25^. Flow cytometry confirmed that PMA treatment significantly upregulated CD11b expression (Fig. 1C and 1D), a hallmark of differentiated macrophages^26^, confirming an M0 macrophage phenotype.

### Reversine-induced CIN in TNBC cells promotes an M2-like phenotype in macrophages in direct co-culture

**Fig. 2:**
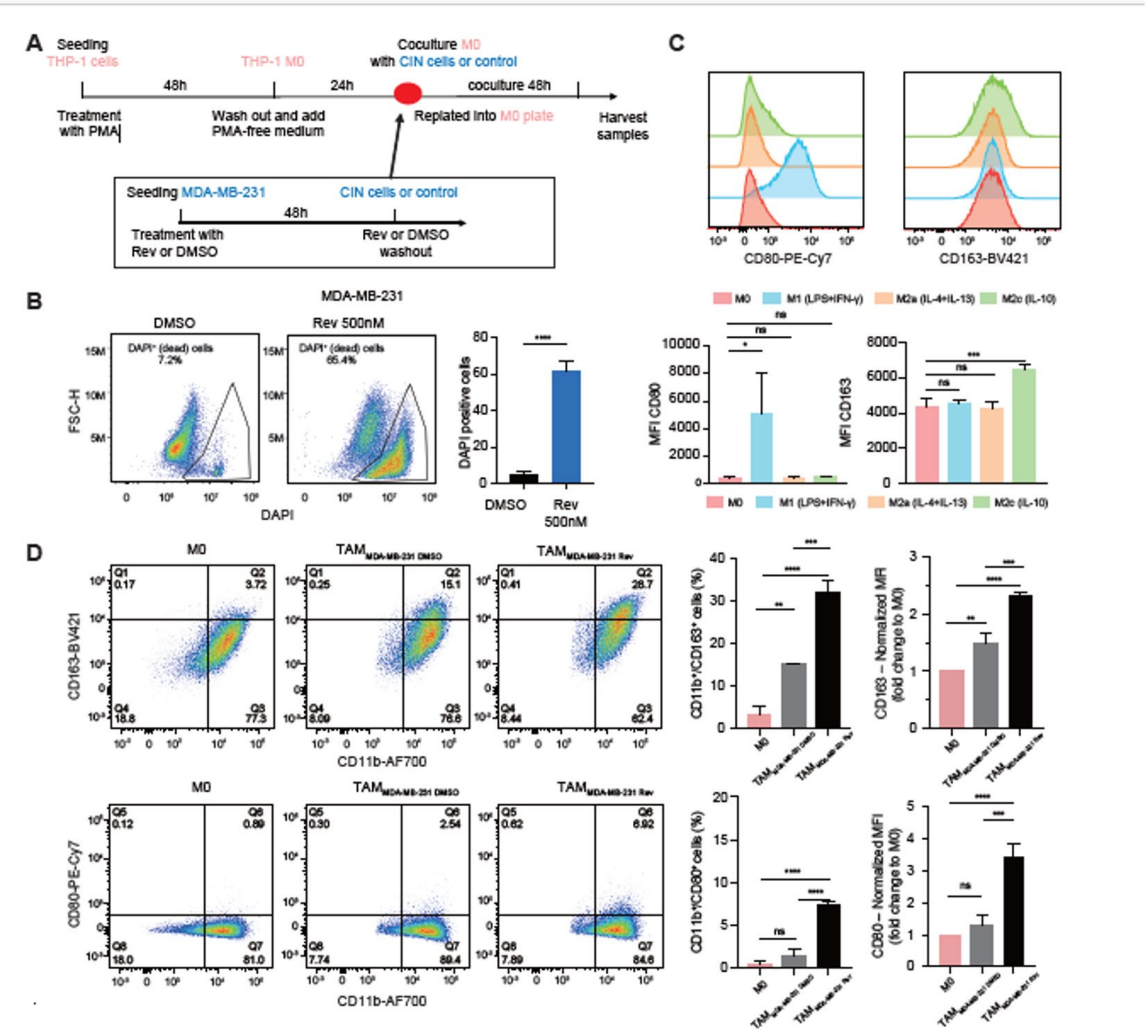
Reversine-treated TNBC cells predominantly induce M2-like polarization of macrophages in direct co-culture. (**A**) Schematic overview of the direct co-culture experiment. MDA-MB-231 cells were pretreated with reversine (Rev, 500 nM) or DMSO for 48 h to induce CIN, followed by 48 h direct co-culture with THP-1-derived M0 macrophages in the same dish. (**B**) Quantification of cancer cell death within 48 h after drug washout, showing an increased proportion of DAPI-positive cells upon reversine treatment. (**C**) Validation of macrophage polarization markers. Representative flow-cytometric histograms and quantification of CD80 and CD163 expression following well-established polarization stimuli (LPS + IFN-γ for M1; IL-4 + IL-13 for M2a; and IL-10 for M2c). (**D**) Flow-cytometric analysis of macrophages after 48 h direct co-culture with control (DMSO) or reversine-treated cancer cells. CD163 (M2) expression was markedly increased, while CD80 (M1) showed a more modest increase, indicating a predominantly M2-like macrophage polarization with limited M1-like features. Right panels show quantification of the percentage of double-positive cells and mean fluorescence intensity (MFI). Data represent the mean ± SD of three biological replicates. Statistical significance for comparisons involving three or more groups was determined using one-way ANOVA followed by Tukey’s multiple comparisons test. Significance levels are indicated as follows: *p < 0.05; **p  < 0.01; ***p < 0.001; ****p < 0.0001.

To investigate whether reversine-treated TNBC cells influence macrophage behavior in a cell-contact-dependent manner, we co-cultured THP-1-derived M0 macrophages with reversine- or DMSO-treated MDA-MB-231 cells for 48 h and analyzed changes in macrophage phenotypes (Fig. 2A)^27,28^. Approximately 60% of reversine-treated cells were non-viable within 48 h after drug washout. Therefore, in addition to direct CIN-mediated effects, CIN-induced cell death may also contribute to the macrophage responses (Fig. 2B). To distinguish macrophages from MDA-MB-231 TNBC cells in our co-culture system, we used CD11b expression (Supplementary Fig. 1).

We then assessed the impact of induced CIN phenotypes on macrophage M1- and M2-like polarization. We first validated M1- and M2-like markers by inducing M1 and M2 phenotypes in M0 macrophages using well-established protocols (LPS + IFN-γ to induce M1 polarization and IL-10 to induce M2 polarization). As expected, CD80 was significantly upregulated following LPS + IFN-γ treatment, while CD163 was elevated following IL-10 treatment, confirming our M1- and M2-like markers (Fig. 2C and Supplementary Fig. 2A)^25^.

We then assessed macrophage polarization in co-cultures with reversine-treated MDA-MB-231 cells, and found a marked increase in CD11b⁺; CD163⁺ (M2-like) macrophages, accompanied by a modest increase in CD11b⁺; CD80⁺ (M1-like) macrophages^29^. This suggests that reversine-treated MDA-MB-231 TNBC cells primarily promote an M2-like phenotype in macrophages with a minority of macrophages adopting an M1-like phenotype (Fig. 2D and Supplementary Fig. 2B).

### Soluble paracrine signaling from reversine-treated TNBC cells drives M2-like polarization in non-contact co-cultures

**Fig. 3:**
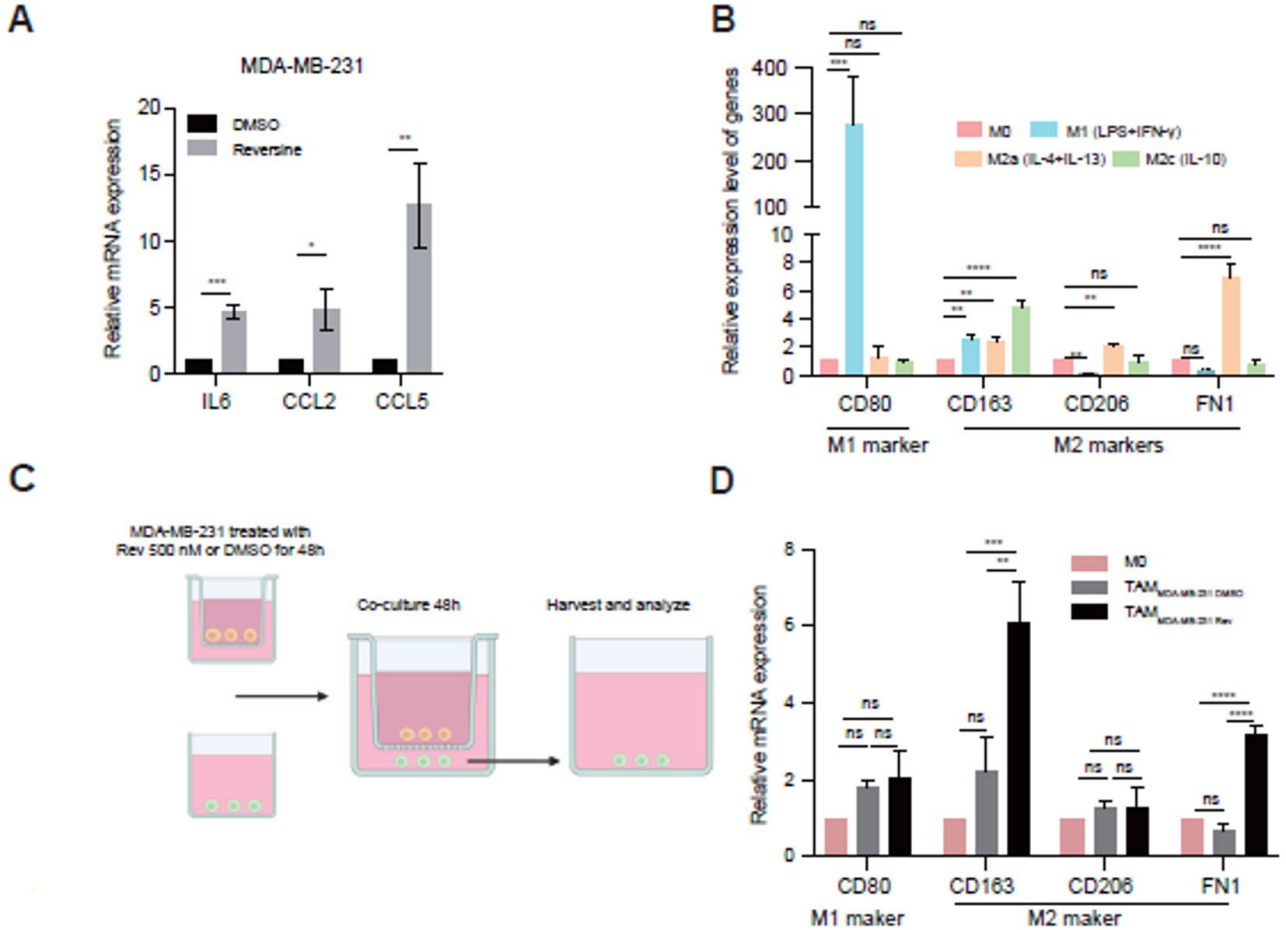
Soluble paracrine signaling from reversine-treated TNBC cells induces M2-like polarization of macrophages in non-contact co-cultures. (**A**) qPCR analysis of the chemokines IL-6, CCL2, and CCL5 in MDA-MB-231 cells treated with reversine (Rev, 500 nM) or DMSO for 48 h, showing elevated expression of proinflammatory cytokines upon reversine treatment. (**B**) Validation of macrophage polarization markers following LPS + IFN-γ (M1), IL-4 + IL-13 (M2a), or IL-10 (M2c) stimuli using expression of CD80 (M1-like markers), CD163, CD206, and FN1 (M2-like markers) by qPCR. (**C**) Schematic overview of non-contact co-cultures. MDA-MB-231 cells pretreated with reversine or DMSO for 48 h were seeded in the upper chamber of a transwell insert, and THP-1-derived M0 macrophages (differentiated with PMA 50 ng/ml for 48 h followed by 24 h of rest) were seeded in the lower chamber. After 48 h of co-culture, macrophages were collected for qPCR analysis. (**D**) Expression of polarization markers in macrophages after non-contact co-culture with DMSO- or reversine-treated TNBC cells, showing that M2-like markers CD163 and FN1 were upregulated in macrophages exposed to soluble factors from reversine-treated cancer cells. Data represent mean ± SD from three independent experiments. Statistical significance for comparisons between two groups was assessed using an unpaired two-tailed Student’s t-test. For comparisons involving three or more groups, one-way ANOVA followed by Tukey’s multiple comparisons test was used. Significance levels are indicated as follows: *p < 0.05; **p  < 0.01; ***p < 0.001; ****p < 0.0001.

As a parallel approach to assess the effect of reversine-treated TNBC cells on macrophage polarization, and to eliminate potential interference from direct cell–cell contact or apoptotic debris from the cancer cells, we also established a non-contact transwell co-culture system^30–32^. For this purpose, we first examined whether reversine treatment induced the expression of macrophage-promoting chemotactic factors in tumor cells. Indeed, qPCR analysis showed that reversine treatment increased expression of CCL2, CCL5, and IL-6 in MDA-MB-231 TNBC cells (Fig. 3A), indicating enhanced chemotactic signaling potential under induced CIN conditions.

As positive controls for polarization markers, we used LPS + IFN-γ to induce M1 polarization, IL-4 + IL-13 to induce M2a polarization, and IL-10 to induce M2c polarization^33^. qPCR results confirmed that CD80 was significantly upregulated under M1 conditions, CD206 and FN1 were increased under M2a conditions, and CD163 was upregulated under M2c conditions as expected (Fig. 3B).

Next, we seeded MDA-MB-231 cells treated with reversine or carrier control into the upper chamber of a transwell, and PMA-differentiated M0 macrophages into the lower chamber. Following 48 h of co-culture, macrophages were harvested for qPCR analysis (Fig. 3C) and assessed for CD80, CD163, CD206, and FN1 expression by qPCR^34^. Macrophages exposed to factors secreted by reversine-treated TNBC cells showed significantly increased expression of the M2-like markers CD163 and FN1, while expression of the M1-like marker CD80 remained unchanged (Fig. 3D). This suggests that paracrine signaling through soluble factors associated with reversine-treated TNBC cells induces macrophage polarization toward an M2-like state, while the minor M1 polarization observed in direct co-cultures might require direct cell–cell contact with the reversine-treated cells or apoptotic debris resulting from CIN-induced cell death.

### Reversine-treated TNBC cells enhance uptake of cancer cell-derived material by macrophages and cancer cell migration

**Fig. 4:**
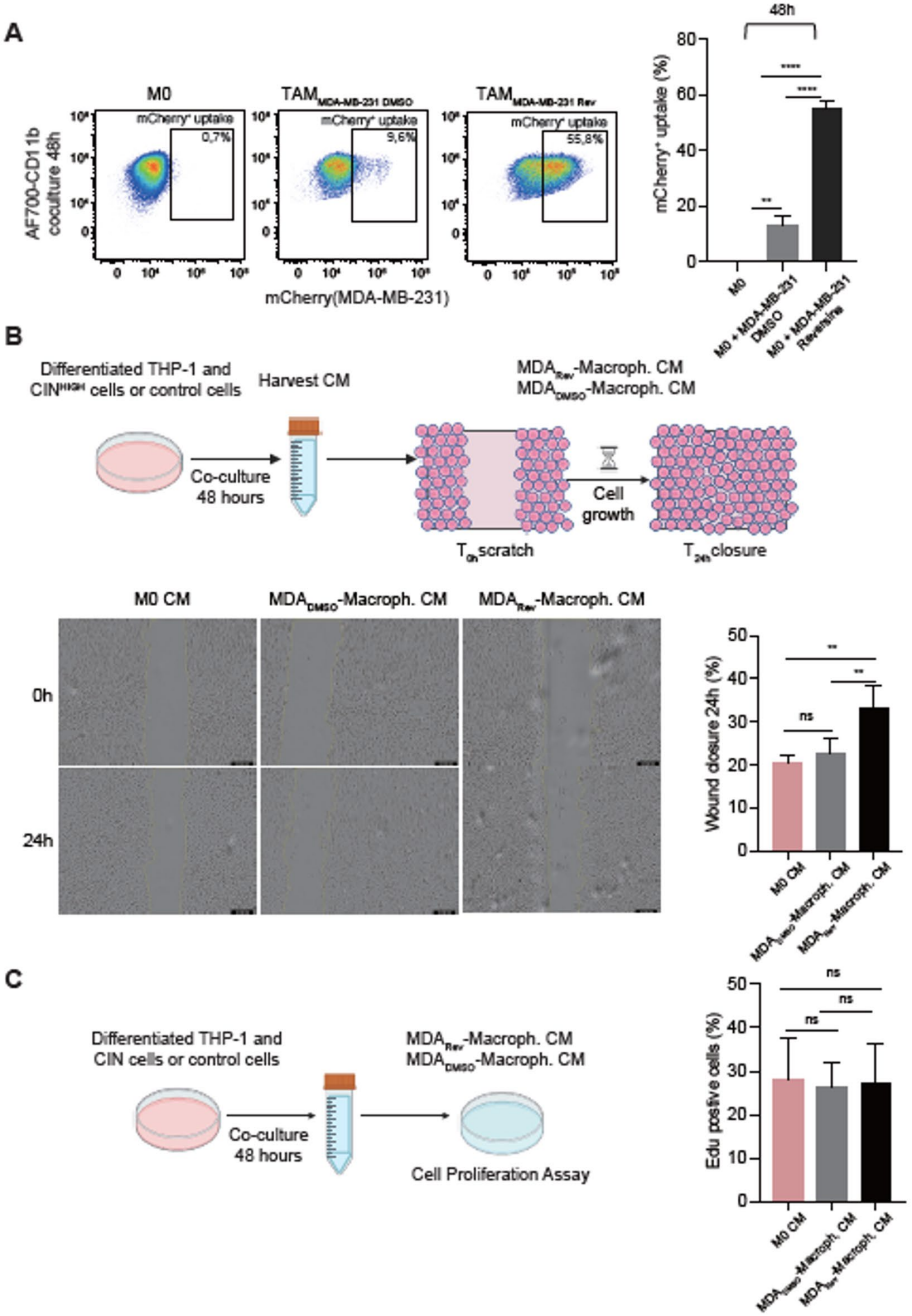
Reversine-treated cancer cells enhance uptake of cancer cell-derived material but not their capacity to promote TNBC cell proliferation. (**A**) Flow-cytometry analysis of co-cultures of reversine- and carrier-treated H2B-mCherry–labeled MDA-MB-231 cells and M0 macrophages. TNBC cells were pretreated with reversine (Rev, 500 nM) or DMSO for 48 h and co-cultured with THP-1–derived M0 macrophages for 48 h. The fraction of mCherry⁺ macrophages was quantified to assess uptake of tumor-derived material. (**B**) Scratch-wound assays evaluating the effect of conditioned media (CM) collected from the co-cultures of carrier-treated or reversine-treated TNBC cells and M0 macrophages. Representative images at 0 h and 24 h and quantification of scratch closure are shown. (**C**) EdU-incorporation assay assessing the effect on proliferation of TNBC cells treated with the same CM as under (B). Data represent the mean ± SD from three biological replicates. Statistical significance for comparisons involving three or more groups was determined using one-way ANOVA followed by Tukey’s multiple comparisons test. Significance levels are indicated as follows: *p < 0.05; **p  < 0.01; ***p < 0.001; ****p < 0.0001.

To further evaluate functional changes in macrophages induced by reversine-treated TNBC cells, we next assessed their capacity to engulf cancer cell-derived material in a direct co-culture system^35^. For this, MDA-MB-231 cells were transduced with H2B-mCherry to fluorescently label their nuclei, treated with carrier or reversine to induce CIN, and co-cultured with M0 macrophages. Flow cytometry analysis of the co-cultures revealed a significant increase in mCherry-positive macrophages in co-cultures with reversine-treated MDA-MB-231 cells (Fig. 4A). It is important to note, however, that given the substantial proportion of non-viable cancer cells in our model system, this increased uptake may in part reflect enhanced engulfment of dying or membrane-compromised cancer cells.

As we observed profound phenotypic changes in macrophages co-cultured with reversine-treated TNBC cells, we next investigated whether TNBC cell behavior was also altered via paracrine signaling in these co-cultures. For this, we collected conditioned medium from co-cultures of THP-1–derived macrophages and MDA-MB-231 cells that had been treated with either reversine or carrier (DMSO) for 48 h, as well as from M0 macrophages cultured alone. Untreated MDA-MB-231 cells were then exposed to these conditioned media, and their migratory capacity was assessed using scratch-wound assays. Indeed, we found that MDA-MB-231 cells exposed to media from co-cultures of reversine-treated MDA-MB-231 cells and M0 macrophages (MDA_Rev_–Macroph. CM) showed increased migration compared to their counterparts treated with media isolated from co-cultures of DMSO-treated MDA-MB-231 cells and M0 macrophages (MDA_DMSO_–Macroph. CM) or media from M0 macrophage monocultures (Fig. 4B). These results suggest that specifically paracrine interactions between macrophages and reversine-treated TNBC cells yield a microenvironment that promotes cancer cell migration^36^. As induced CIN also promotes cell death, we cannot fully rule out that factors secreted by dying cells as a result of CIN also contribute to this phenotype.

To determine potential effects of the co-cultured cells on the proliferation of non-exposed cancer cells, we performed EdU incorporation assays. Here, treatment with conditioned medium from co-cultures of reversine-treated MDA-MB-231 cells and M0 macrophages (MDA_Rev_–Macroph. CM) did not alter EdU incorporation of MDA-MB-231 cells compared to MDA-MB-231 cells treated with conditioned medium isolated from co-cultures of DMSO-treated MDA-MB-231 cells and M0 macrophages (MDA_DMSO_–Macroph. CM) or conditioned media from M0 monocultures (Fig. 4C). These results indicate that paracrine factors released by TNBC cells with CIN or exposed macrophages do not alter proliferation of recipient cancer cells.

In conclusion, our work shows that drug-induced CIN phenotypes in TNBC cells promote macrophage reprogramming, predominantly towards an M2-like phenotype, with some macrophages adopting M1-like features, the latter only in direct co-cultures. Together, the observed increase in migration potential of TNBC cells exposed to conditioned media from co-cultures of reversine-treated TNBC cells and macrophages, our findings suggest that drug-induced CIN can have profound effects on both macrophages as well as cancer cells within the same microenvironment.

## Discussion

Our work reveals that acute CIN phenotypes induced in TNBC cells polarize macrophages toward an M2-like phenotype, with a minority of macrophages adopting an M1-like phenotype. Macrophage polarization is likely driven by chemokines secreted by reversine-treated TNBC cells, although further work is required to identify the exact factors secreted by the cancer cells that drive macrophage polarization.

Our work aligns well with previous pan-cancer studies showing that somatic copy number alterations (SCNAs) can influence macrophage polarization^9^. Amplifications of chromosomes 2, 20q, and 22q (harboring genes such as CTLA4, CD40, and ADORA2A) and deletions in 5q, 9p, and 19 (including IL13, IFNA1/2, and ICAM1) have been associated with changes in macrophage states^14^. However, the effect of CIN^HIGH^ cancer cells on macrophages appears to be tumor-type and context-dependent. For instance, in melanoma models, acute CIN has been shown to activate the CD47–SIRPα pathway promoting M1-mediated antitumor immunity^37^, while in HER2-positive breast cancer, chronic CIN induced by Plk1 inhibition promotes the accumulation of M2-type immunosuppressive macrophages^38^.

We also observed enhanced uptake of tumor-derived material from reversine-treated TNBC cells by macrophages. This increased uptake may, at least in part, reflect the clearance of dying or membrane-compromised tumor cells, given the high proportion of non-viable cells in this experimental setting. Therefore, we cannot exclude that increased cell death also contributes to the observed phagocytic phenotype. While we did not identify the precise mechanism triggering phagocytosis in this setting, others have reported that signals derived from dying tumor cells activate STING-dependent type I interferon responses in macrophages, thereby contributing to antitumor adaptive immunity^21,39^. However, despite their increased uptake of cancer cell-derived material, macrophages exposed to reversine-treated cancer cells remained skewed toward an immuno-suppressive M2 phenotype, suggesting that increased phagocytic activity does not necessarily equal immune activation. Indeed, macrophage-mediated clearance of dying cells (efferocytosis) is well known to promote anti-inflammatory and immunosuppressive responses. During efferocytosis, macrophages suppress pro-inflammatory signaling while upregulating anti-inflammatory mediators such as IL-10 and TGF-β, thereby promoting immune tolerance^40,41^. In a tumor context, M2-like tumor-associated macrophages (TAMs) can efficiently clear dying cells, preventing the accumulation of extracellular cGAMP and ATP, and thus suppressing immune activation^42^. This process is mediated by receptors such as MerTK. Therefore, blocking MerTK-mediated efferocytosis may help prolong STING stimulation and restore immune activity^21^. Furthermore, even if cGAS–STING is triggered in tumor cells, downstream immune signaling may be negatively regulated by pathways like p38 MAPK^43^, further dampening immune-stimulating M1 responses. However, further work is required to test these predictions in cell models with induced and chronic CIN.

Our study also has some limitations. First, our observations are based on *in vitro* co-culture models, which do not fully recapitulate the complexity of the *in vivo* tumor microenvironment. Second, the molecular mechanisms underlying macrophage polarization induced by reversine-treated cancer cells remain to be fully uncovered. Third, our model system makes use of reversine, which, while being an accepted tool to induce CIN, might still have effects beyond CIN. Fourth, alternative drivers of CIN may differentially shape cellular and immune responses. Future studies using orthogonal approaches will be important to validate and build upon these findings. Finally, macrophage polarization was primarily assessed at the transcriptional level. Given that cell signaling also heavily relies on post-translational regulation, (phospho-) proteomics assessment will be required to better map the signaling underlying these phenotypes.

Overall, our findings suggest that drug-induced CIN, an established driver of (therapy-induced) tumor heterogeneity and evolution, promotes changes in the microenvironment through its impact on macrophages. The combined effect of increased chemokine expression and M2 polarization may contribute to immune evasion and resistance to immunotherapy observed in CIN^HIGH^ cancers^44^. Another potential implication of our work is that commonly applied anti-cancer drugs that induce CIN (*e.g.* Paclitaxel or Vincristine)^45^ might promote similar phenotypes *in vivo* and thus contribute to immune evasion and metastasis. Therefore, targeting CIN-associated immunosuppressive pathways or reprogramming TAMs—for example, via STAT3 or MerTK inhibition—may represent potential therapeutic strategies to target CIN-associated tumor–immune interactions, particularly when combined with CIN-inducing drugs.

## Materials and methods

### Cell culture

MDA-MB-231 cells and 293FT cells were cultured in Dulbecco’s Modified Eagle Medium (DMEM) (Gibco, 31966–021) supplemented with 10% fetal bovine serum (FBS) (Thermo Fisher Scientific, 11573397) and 1% penicillin/streptomycin (P/S) (Gibco, 15140–122). The THP-1 cell line was cultured in RPMI 1640 medium with GlutaMAX supplement (Gibco, 72400–054) supplemented with 10% FBS and 1% P/S. All cell lines were obtained from the American Type Culture Collection (ATCC). All cell lines were grown at 37 °C and 5% CO_2_. For passaging, cells were detached using TrypLE Express (Gibco, 12605–010).

### Lentiviral transduction

Lentiviral transductions of pIGPZ–H2B–mCherry and other constructs were performed by transfecting 293FT cells with 3 μg of the desired plasmid together with 3 μg psPAX2 and 1 μg pMD2.G (Addgene #12260 and #12259; gifts from Didier Trono). The pIGPZ–H2B–mCherry plasmid was generated by subcloning H2B–mCherry from an existing construct using NotI and BsrGI (Bioke) restriction sites. Viral supernatants were collected 48–72 h post-transfection, filtered through a 0.45-µm membrane (TPP, Y99745), and transferred onto target cells in the presence of 8 μg/ml polybrene (Millipore, TR-1003-G) to enhance transduction efficiency.

### Extraction of RNA and qRT-PCR

For quantitative PCR (qPCR) analysis, total RNA was isolated using the RNA Plus Isolation Kit (Qiagen, 74136). Complementary DNA (cDNA) was synthesized from 1.5 µg of RNA using the LunaScript RT SuperMix Kit (NEB, M3010X) in a total reaction volume of 20 µL. qPCR was carried out with iTaq Universal SYBR Green Supermix (Biorad, 1725124) on a LightCycler® 480 real-time PCR system (Roche). Primer sequences are provided in Supplementary Table 1. Relative expression levels were calculated using the ΔΔCt method, and data visualization and statistical analyses were performed with Microsoft Excel and GraphPad Prism.

### Direct co-culture assay

THP-1 cells were seeded in 6-well plates at a density of 1 × 10⁶ cells per well and treated with 50 ng/ml PMA (Sigma-Aldrich, P1585) for 48 h to induce differentiation into M0 macrophages. After treatment, the cells were washed gently with PBS and then rested for 24 h in fresh PMA-free RPMI-1640 complete medium. MDA-MB-231 cells were cultured in 6 cm dishes and treated with 500 nM Reversine (Sigma, R3904) or DMSO (ITW Reagents, A3672,0250) for 48 h. After treatment, cells were washed with PBS to remove residual drug, trypsinized, counted, and added directly to the THP-1 macrophages at a 1:1 ratio. Co-culture was performed in RPMI-1640 medium for 48 h. Following co-culture, THP-1 cells were collected for downstream phenotypic and functional analyses.

### Non-contact transwell co-culture of human TNBC cells and THP-1-derived macrophages

THP-1 monocytes were seeded into the lower chambers of 6-well Transwell plates (0.4 μm pore size, cellqart, 9300402) and treated with 50 ng/ml PMA for 48 h to induce macrophage differentiation. Afterward, cells were washed with PBS (Thermo Fisher Scientific, 12037539) and rested in PMA-free medium for 24 h. MDA-MB-231 TNBC cells were seeded in 6 cm dishes and treated with 500 nM Reversine or DMSO for 48 h. Cells were then trypsinized, counted, and seeded into the upper Transwell inserts at a 1:1 ratio with THP-1 cells. The inserts were placed above the pre-differentiated THP-1 macrophages, allowing indirect (non-contact) co-culture for 48 h in RPMI-1640 medium with 10% FBS. After co-culture, THP-1 cells were collected for further analysis.

### Time-lapse imaging

Chromosome segregation errors were monitored by live-cell time-lapse microscopy. To visualize chromosomes, cell lines were transduced with lentiviral H2B–mCherry constructs. MDA-MB-231 cells were seeded into glass-bottom imaging dishes (Greiner Bio-One, #627870) one day before imaging. Live imaging was carried out on a DeltaVision Elite microscope (GE Healthcare) equipped with a CoolSNAP HQ2 camera and a 40 × /0.6 NA immersion objective (Olympus). Images were acquired every 6 min over a period of at least 20 h, with each stack comprising 30–40 z-sections spaced 0.5 µm apart. Image sequences were processed and analyzed using ICY software (Institut Pasteur). Only mitotic cells were included in the quantification. For drug-treated conditions, cells were exposed to the compound one hour before the start of imaging.

### EdU incorporation assay

For proliferation analysis, 20,000 cells were seeded on glass coverslips in 12-well plates. The following day, cells were incubated with 10 µM 5-ethynyl-2′-deoxyuridine (EdU; Sigma-Aldrich, 900584) for 24 h. After incubation, cells were fixed with 4% formaldehyde solution (diluted from 37–41% formaldehyde stock; Thermo Fisher Scientific, F/1501/PB15) in PBS and subjected to a click-chemistry reaction containing 2 mM CuSO₄, 4 µM sulfo-Cy3-azide (Lumiprobe, cat. no. D1330), and 20 mg/ml sodium ascorbate in PBS. Images were captured using an Olympus fluorescence microscope and analyzed with ImageJ software.

### *In Vitro* uptake of tumor-derived material assay

THP-1 cells were seeded in 6 cm dishes (1 × 10⁶ cells/well) and treated with 50 ng/ml PMA for 48 h to induce differentiation into M0 macrophages. Cells were then washed with PBS and rested for 24 h in PMA-free RPMI-1640 medium. MDA-MB-231 cells were transduced with H2B-mCherry to label nuclei and treated with 500 nM Reversine or DMSO for 48 h. After treatment, tumor cells were washed with PBS to remove residual drug, trypsinized and added directly to M0 macrophages at a 1:1 ratio. Co-culture was performed in RPMI-1640 medium at 37 °C for 48 h. After incubation, wells were washed twice with PBS to remove non-engulfed tumor cells. Cells were collected and analyzed using a Quanteon flow cytometer (NovoCyte, Agilent). CD11b staining was used to identify macrophages, as THP-1–derived macrophages are CD11b⁺, and MDA-MB-231 cells are CD11b⁻. The uptake rate was calculated as the percentage of CD11b⁺H2B-mCherry⁺ macrophages among total CD11b⁺ cells.

### Flow cytometry

For flow cytometry assessment, single-cell suspensions were washed with 1 × PBS and stained in 1 × PBS containing 2% FBS and the relevant surface antibodies (Table S2) for 30 min at 4 °C. After staining, cells were washed again with PBS and resuspended in 300 µL of PBS for acquisition. Low-concentration DAPI (Sigma, D9542) was added immediately before acquisition (5 min) and analyzed without washing to detect membrane-compromised non-viable cells. Samples were analyzed on a BD flow cytometer (BD Biosciences), and data were processed using FlowJo software (v10.x; Tree Star Inc.).

### Scratch assays

For migration analysis, untreated MDA-MB-231 cells were seeded into 12-well plates and cultured until confluence. A straight scratch was made across the cell monolayer using a 1-mm pipette tip, followed by two gentle PBS washes to remove detached cells. Cells were then incubated in conditioned media (CM) collected from different experimental groups, including M0 CM, DMSO- and Reversine-treated co-culture CM (MDA_DMSO_–Macroph. CM and MDA_Rev_–Macroph. CM), under serum-free conditions. Scratch closure was monitored using an IncuCyte live-cell imaging system at 10 × magnification, with images captured every 2 h for 24–48 h. The wound area was quantified using Fiji (ImageJ 1.53c), and migration rates were calculated as the percentage of wound closure relative to the initial wound area.

### Statistical analysis

All quantitative data are presented as mean ± standard deviation (SD) from at least three independent experiments. Comparisons between two groups were performed using a two-tailed Student’s t-test, while comparisons among multiple groups were analyzed by one-way ANOVA followed by Tukey’s post-hoc test. Statistical analyses were conducted using GraphPad Prism (version 9.5.1). A p-value < 0.05 was considered statistically significant and denoted as p < 0.05 (*), p < 0.01 (**), p < 0.001 (***), and p < 0.0001(****).

## Supplementary Information

Supplementary Information 1.

Supplementary Information 2.

Supplementary Information 3.

## Data Availability

All data generated or analyzed in this study are included in this article and its Supplementary Information files.
