# Supplementary material for: Induced chromosomal instability in triple negative breast cancer cells promotes M2-like polarization of macrophages
Source: Sci Rep. 2026 Jun 6;16:25782. doi: 10.1038/s41598-026-55242-0 (PMC13482341; doi:10.1038/s41598-026-55242-0)
Supplement: Supplementary file 1 [file 41598_2026_55242_MOESM1_ESM.pdf]

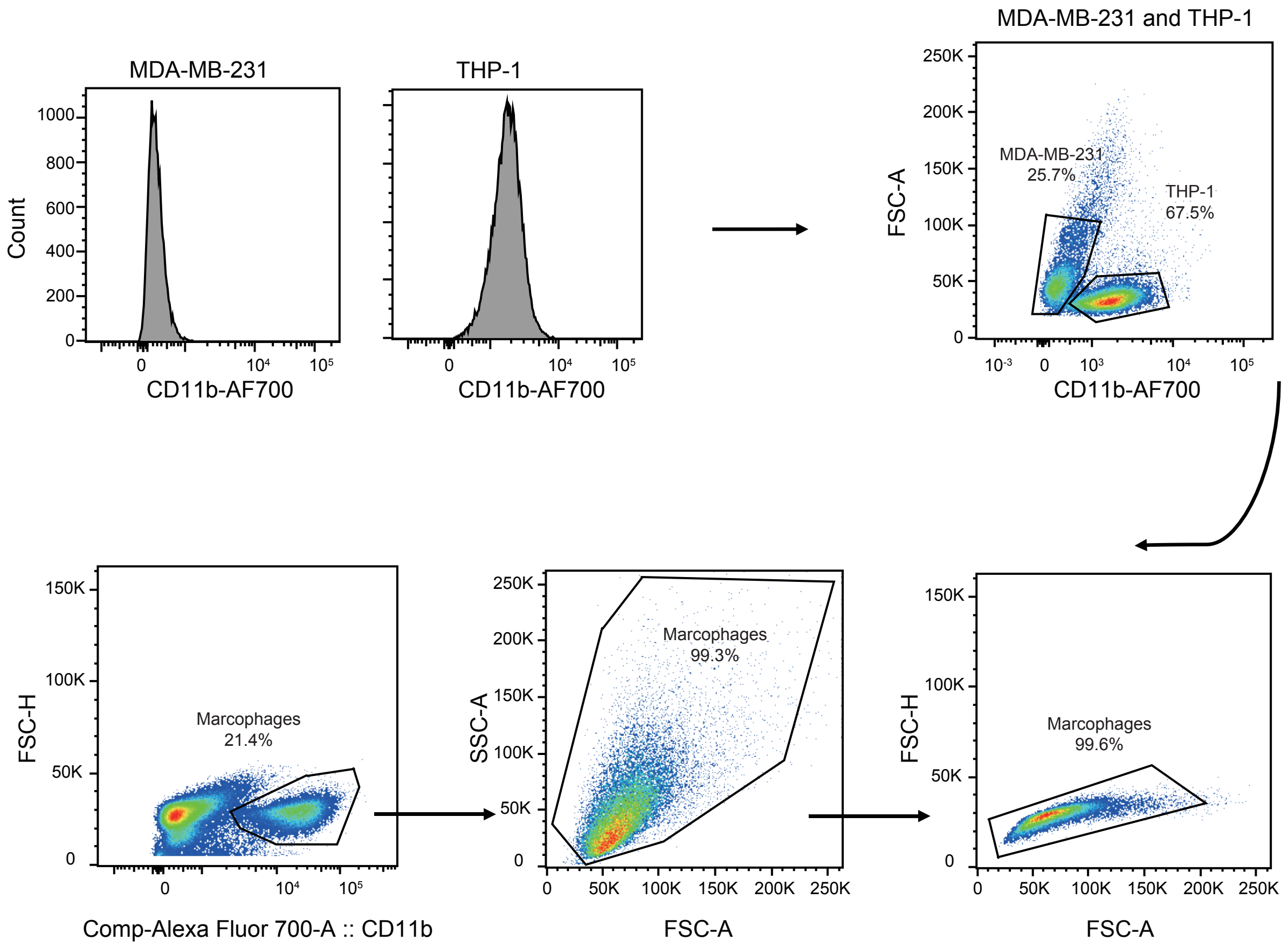

Tian et al, Sup. Fig. 1
